# Mechanistic and Clinical Differences Between Daratumumab and Isatuximab in Multiple Myeloma: Emerging Roles of 1q Gain and Immune Remodeling

**DOI:** 10.3390/cells15151331

**Published:** 2026-07-24

**Authors:** Jiro Kikuchi, Hiroshi Yasui

**Affiliations:** 1 Division of Emerging Medicine for Integrated Therapeutics (EMIT), Center for Molecular Medicine, Jichi Medical University, Shimotsuke 329-0498, Japan; kiku-j@jichi.ac.jp; 2 Department of Hematology and Oncology, St. Marianna University School of Medicine, Kawasaki 216-8511, Japan; 3 The Institute of Medical Science, The University of Tokyo, Tokyo 108-8639, Japan

**Keywords:** multiple myeloma, daratumumab, isatuximab, CD38, anti-CD38 antibody, mechanism of action, 1q gain, immune remodeling, tunneling nanotubes, extramedullary disease

## Abstract

**Highlights:**

**What are the main findings?**

- Daratumumab and isatuximab exhibit distinct biological mechanisms beyond their shared CD38 targeting.
- 1q gain and the PBX1–FOXM1 axis may predict enhanced sensitivity to isatuximab.

**What is the implication of the main findings?**

- Daratumumab and isatuximab differentially remodel the immune microenvironment and residual disease.
- Biology-guided selection of anti-CD38 antibodies may optimize treatment sequencing in MM.

**Abstract:**

Anti-CD38 monoclonal antibodies have substantially improved outcomes in multiple myeloma (MM). Although daratumumab and isatuximab target the same antigen, accumulating evidence indicates that they differ in epitope recognition, biological activity, and immunomodulatory properties, suggesting these agents may not be therapeutically interchangeable. This review summarizes the molecular and immunological mechanisms underlying their distinct antitumor effects and their implications for treatment selection. Isatuximab binds near the catalytic site of CD38, resulting in potent enzymatic inhibition, enhanced antibody internalization, FOXM1 suppression, and reactive oxygen species-mediated cytotoxicity, which may preferentially target MM cells harboring 1q21 amplification. In contrast, daratumumab exerts prominent Fc-dependent immune effects, including trogocytosis-mediated downregulation of CD38 and VLA-4, suppression of cell adhesion-mediated drug resistance, and modulation of the immune microenvironment, potentially enhancing subsequent T-cell-redirecting therapies. We further discuss the relevance of these mechanistic differences to measurable residual disease, extramedullary disease, and sequencing with BCMA- and GPRC5D-directed immunotherapies. Finally, we propose a biology-guided treatment-selection model integrating genomic alterations, tumor biology, and immune remodeling to support precision medicine for patients with MM.

## 1. Introduction

Multiple myeloma (MM) remains largely incurable despite significant advances in treatment over the past two decades. The incorporation of anti-CD38 monoclonal antibodies into frontline and relapsed/refractory MM (RRMM) treatment has substantially improved patient outcomes [1]. Daratumumab and isatuximab are now widely used across multiple treatment settings, including newly diagnosed MM (NDMM) and RRMM. Recent clinical guidelines have incorporated quadruplet regimens containing either antibody for both transplant-eligible and transplant-ineligible patients. However, despite targeting the same antigen, the optimal selection between daratumumab and isatuximab remains uncertain.

Although both antibodies share the same molecular target, accumulating evidence from basic and clinical research suggests that their mechanisms of action, efficacy profiles, and clinical applications differ in important ways. In particular, amplification of the chromosome 1q21 region (1q gain), the most common cytogenetic abnormality in MM [2], has recently emerged as a potential determinant of differential sensitivity to anti-CD38 antibodies [3,4]. Understanding these differences is essential for optimizing treatment selection in individual patients.

This review summarizes the current understanding of the biological and clinical differences between daratumumab and isatuximab and proposes a mechanistic framework linking 1q-associated disease biology, immune microenvironment remodeling, and the selection of anti-CD38 antibodies.

## 2. CD38: Biology and Cellular Function

### 2.1. Structure and Expression

CD38 is a type II transmembrane glycoprotein expressed at high levels on MM plasma cells and at variable levels on normal lymphoid and myeloid cells. Its expression pattern makes it an attractive therapeutic target. CD38 is a type II single-pass transmembrane protein of approximately 300 amino acids, comprising a short N-terminal cytoplasmic tail, a single transmembrane domain, and a large C-terminal extracellular domain that harbors the enzymatic active site [5]. CD38 surface expression is markedly elevated on MM plasma cells compared with normal hematopoietic progenitors, while expression on normal bone marrow plasma cells is even higher. This differential expression pattern defines the therapeutic window of anti-CD38 antibodies [6]. Importantly, CD38 surface density on MM cells is dynamically regulated: all-trans retinoic acid (ATRA) and lenalidomide upregulate CD38 expression, potentially augmenting susceptibility to anti-CD38 antibody-mediated cytotoxicity [7]. Conversely, prolonged anti-CD38 antibody exposure progressively downregulates CD38 surface expression, constituting a principal mechanism of acquired resistance [8].

### 2.2. Enzymatic Activity and Signaling

**Figure 1:**
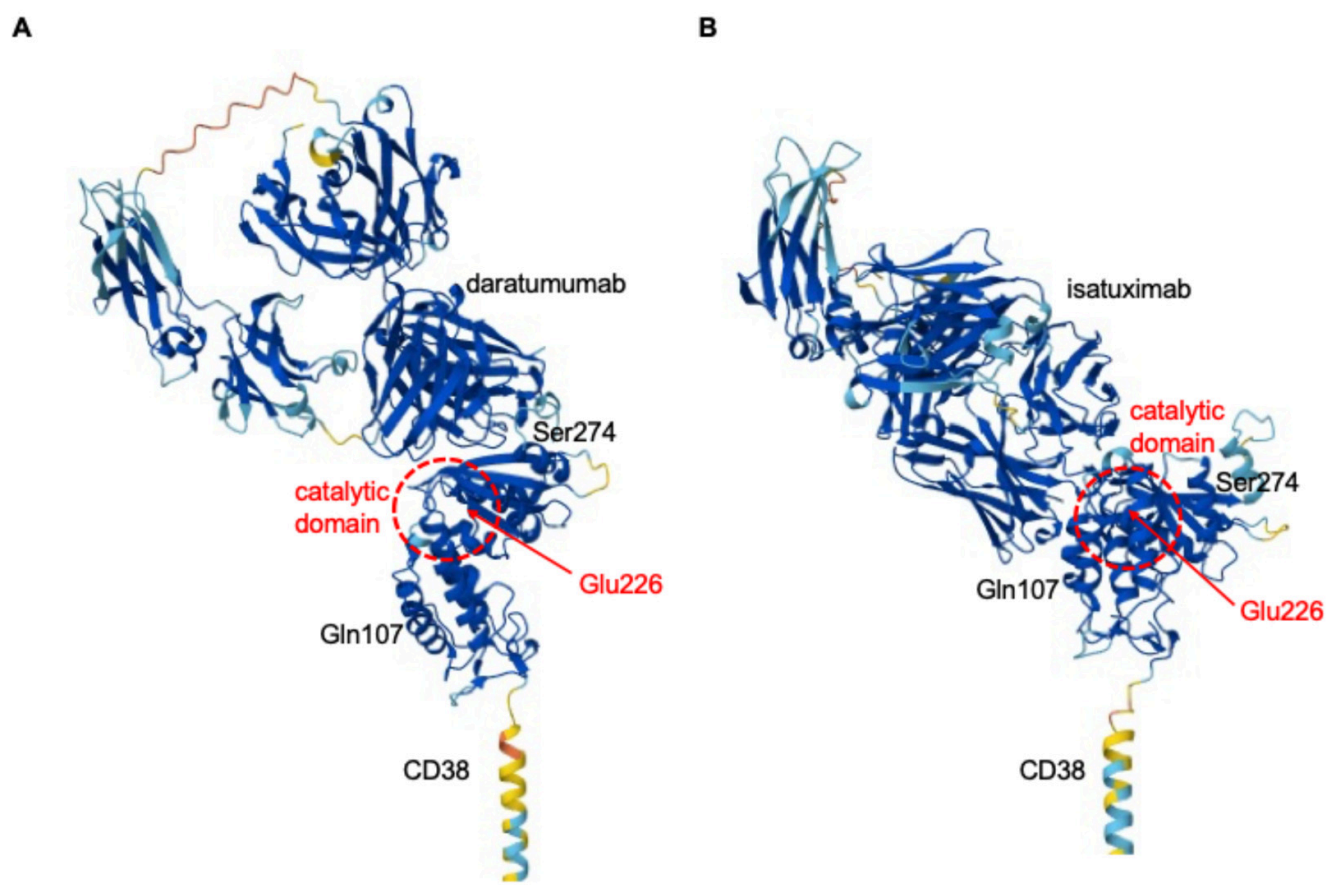
Structural basis for the differential mechanisms of daratumumab and isatuximab. Binding mode of daratumumab (**A**) and isatuximab (**B**) on the extracellular domain of CD38 predicted by AlphaFold 3. Structural models were generated using the full-length human CD38 amino acid sequence (RefSeq: NP_001766.2) and the amino acid sequences of daratumumab (Kyoto Encyclopedia of Genes and Genomes [KEGG] DRUG: D10777) and isatuximab (KEGG DRUG: D11050). Structural predictions were performed using the default AlphaFold 3 settings without applying experimental structural restraints or custom templates. Representative antibody-contact residues (Ser274 for daratumumab and Gln107 for isatuximab) were identified according to the predicted hydrogen-bonding interactions in the AlphaFold 3 model and are shown for illustrative purposes. In addition, the key amino acid residue involved in CD38 enzymatic activity (Glu226) is indicated. Residue numbering follows the full-length human CD38 sequence (RefSeq: NP_001766.2). Red circles highlight the catalytic domain of CD38.

CD38 functions as a multifunctional ectoenzyme with both cyclase and hydrolase activities, catalyzing the synthesis of cyclic ADP-ribose (cADPR) and ADPR from NAD+ [9]. These products regulate intracellular calcium mobilization and downstream signaling pathways critical for cell survival and proliferation. Specifically, CD38 mediates at least three distinct enzymatic reactions: (1) cyclase activity, converting NAD+ to cADPR, which releases Ca^2+^ from ryanodine receptor-gated endoplasmic reticulum stores; (2) hydrolase activity, degrading NAD+ or cADPR to ADP-ribose (ADPR); (3) base-exchange activity, producing nicotinic acid adenine dinucleotide phosphate (NAADP) from NADP+, which mobilizes Ca^2+^ from lysosomal stores [10,11]. Through sustained NAD+ consumption, CD38 modulates the cellular NAD+/NADH redox ratio, influencing mitochondrial metabolism and sirtuin-mediated epigenetic regulation in MM cells [5]. Notably, isatuximab, but not daratumumab, directly inhibits CD38 cyclase activity by engaging an epitope that occludes the catalytic domain (Figure 1), a property that may contribute to its unique lysosome-mediated direct cytotoxicity, independent of immune effector functions (Table 1) [12]. Given that cADPR functions as a key intracellular calcium-mobilizing messenger [13], isatuximab-mediated inhibition of CD38 enzymatic activity may potentially modulate calcium-dependent signaling pathways in MM cells. The precise biological significance of this mechanism and its contribution to the differential clinical effects of isatuximab and daratumumab in multiple myeloma, however, remain to be fully elucidated.

### 2.3. CD38 in the Bone Marrow Microenvironment (BMME)

CD38 is expressed on MM cells, immune effector cells, and stromal cells within the BMME. Consequently, anti-CD38 antibodies have complex effects on both tumor cells and the immune milieu. A critical immunological consequence of this broad CD38 expression is the antibody-mediated killing of CD38+ NK cells. NK cells, which serve as principal ADCC effectors, co-express CD38 at moderate levels and are thus susceptible to elimination by daratumumab, resulting in an early decline in circulating NK cell counts [14]. Nevertheless, residual NK cells adopt an activated phenotype with augmented cytotoxic capacity, and NK cell numbers recover during the course of treatment [14]. In parallel, anti-CD38 antibodies deplete CD38+ immunosuppressive populations, including regulatory T cells (CD38+ Tregs) and myeloid-derived suppressor cells (MDSCs), thereby facilitating antitumor T-cell expansion and broadening of the T-cell receptor repertoire [15]. Daratumumab and isatuximab differ in the magnitude of these effects: daratumumab more potently eradicates Tregs, whereas isatuximab preferentially suppresses Treg activity and additionally inhibits tumor-associated macrophages (TAMs), as summarized in Table 2.

## 3. Mechanisms of Action: Daratumumab vs. Isatuximab

### 3.1. Epitope Binding and Structural Differences

Daratumumab and isatuximab bind to distinct, yet partially overlapping, epitopes on the CD38 extracellular domain (Figure 1) [16]. These structural differences in epitope binding underlie their differential functional activities (Table 1 and Table 2).

### 3.2. Fc-Mediated Effector Functions

#### 3.2.1. ADCC

Both daratumumab and isatuximab mediate ADCC through engagement of FcγRIIIa on NK cells and macrophages, leading to MM cell killing.

#### 3.2.2. Complement-Dependent Cytotoxicity (CDC)

A key mechanistic difference is that daratumumab potently induces CDC, whereas isatuximab exhibits minimal CDC activity. This difference may be attributable to the distinct epitopes engaged and the resulting conformational changes in the Fc region.

#### 3.2.3. Antibody-Dependent Cellular Phagocytosis (ADCP)

Both antibodies promote ADCP, contributing to the elimination of MM cells by macrophages in the bone marrow.

### 3.3. Direct Cell Death Induction

Beyond Fc-mediated mechanisms, isatuximab has been shown to directly induce apoptosis in MM cells, independent of immune effector cells [17]. Recent work by Kikuchi et al. demonstrated that isatuximab downregulates FOXM1, a transcription factor that promotes cell survival, in MM cells with 1q+ aberrations, thereby triggering direct cell death [18].

### 3.4. Immunomodulatory Effects

Anti-CD38 antibodies deplete CD38-expressing immunosuppressive cells, including regulatory T cells (Tregs), regulatory B cells (Bregs), and MDSCs, thereby restoring antitumor immune function [14].

### 3.5. CD38 Enzymatic Inhibition

The catalytic center of CD38 is located within its extracellular domain, with Glu226 serving as the key catalytic residue responsible for NAD hydrolysis and cyclic ADP-ribose generation [19]. Isatuximab, but not daratumumab, directly inhibits the enzymatic activity of CD38 [12]. Structural studies have demonstrated that isatuximab binds to an epitope located in close proximity to the catalytic pocketof CD38, involving residues such as Glu226. In contrast to daratumumab, whose epitope is located farther from the catalytic center, the proximity of the isatuximab epitope to the enzymatic site provides a structural basis for its direct inhibition of CD38 NADase activity (Figure 1).

### 3.6. Effects on Cell Adhesion-Mediated Drug Resistance

Accumulating evidence indicates that the eradication of minimal residual disease (MRD) is a critical determinant of long-term survival in MM. Deep responses leading to sustained MRD negativity have consistently been associated with prolonged progression-free survival (PFS) and overall survival (OS) across multiple therapeutic settings [20]. Biologically, MRD cells are thought to persist within specialized hypoxic niches of the BMME, where interactions with bone marrow stromal cells confer resistance to therapeutic agents. This phenomenon, commonly referred to as cell adhesion-mediated drug resistance (CAM-DR), represents a major obstacle to complete disease eradication [21]. Transcriptomic analyses of residual MM cells have demonstrated the upregulation of multiple adhesion molecules, suggesting that enhanced adhesive properties are a hallmark of MRD biology. Among these molecules, very late antigen-4 (VLA-4; integrin α4β1, CD49d/CD29) has emerged as a key mediator of MM cell retention within the bone marrow niche [22]. VLA-4 facilitates adhesion to stromal cells and extracellular matrix components, thereby activating prosurvival signaling pathways and protecting MM cells from therapeutic stress [2,3,4,5,6,7,8,9,10,11,12,13,14,15,16,17,18,19,20,21,22,23,24,25,26].

Among key anti-MM drugs, bortezomib has been shown to reduce VLA-4 expression on MM cells and partially overcome adhesion-mediated resistance, thereby contributing to its ability to eradicate residual tumor populations within the BMME [27,28]. A similar mechanism may also contribute to the activity of daratumumab. In addition to its established immune-mediated effects, daratumumab induces trogocytosis by macrophages, resulting in the removal of CD38-containing membrane fragments from MM cells. Importantly, this process is accompanied by the simultaneous reduction of several surface proteins located within the same membrane domains, including CD49d and CD29 [29]. Consequently, daratumumab-mediated trogocytosis may reduce VLA-4 expression and impair the adhesive interactions that support MRD persistence. Taken together, these observations suggest that daratumumab may facilitate the elimination of residual disease and contribute to the achievement of durable MRD by disrupting adhesion-mediated survival signals within the BMME (Table 2).

## 4. The Emerging Clinical Importance of 1q Gain

Amplification of the chromosome 1q21 region (1q gain) is observed in approximately 35–50% of NDMM and becomes increasingly prevalent during disease progression and relapse [2]. Recent genomic studies have demonstrated that the presence of 1q gain in addition to conventional high-risk cytogenetic abnormalities substantially worsens PFS and OS. Importantly, 1q gain frequently coexists with del(17p), t(4;14), t(14;16), or 1p deletion, creating biologically aggressive “double-hit” disease [30].

Single-cell transcriptomic analyses comparing paired diagnostic and relapsed samples have provided further insight into the biological significance of 1q gain [31]. Genes located within the 1q region represent the most consistently upregulated genomic cluster during disease evolution and relapse, suggesting that this lesion plays a central role in treatment resistance and clonal selection. Several genes within the 1q region have been implicated in disease progression, including MCL1, SLAMF7, and proteasome subunits. These observations suggest that 1q gain is not merely a prognostic marker but may represent a fundamental biological driver of relapse.

### 4.1. Soluble SLAMF7 and the Immune-Suppressive 1q Signature

Among genes amplified within chromosome 1q, SLAMF7 has attracted particular attention due to its dual role in MM biology and immune regulation. SLAMF7 is highly expressed on myeloma cells and several immune cell populations, including NK cells and macrophages [32,33]. A soluble form of SLAMF7 (sSLAMF7) has been identified in the serum of patients with MM, but not in healthy individuals. Elevated serum levels of sSLAMF7 are associated with inferior clinical outcomes and enhanced tumor proliferation [33,34,35,36]. Although originally believed to arise through proteolytic cleavage of membrane-bound SLAMF7, subsequent studies have suggested that sSLAMF7 may instead originate from alternatively spliced transcripts lacking the transmembrane domain [37].

Experimental studies have demonstrated that sSLAMF7 functions as an autocrine and paracrine growth factor, promoting MM cell proliferation. Moreover, sSLAMF7 stimulates macrophages to produce inflammatory cytokines such as TNF-α and IL-6, thereby facilitating macrophage expansion [38]. Recent single-cell analyses of RRMM have revealed substantial accumulation of TAMs and the generation of exhausted T cells within the BMME [31]. TAMs express SLAMF7 and interact directly with SLAMF7-positive CD8+ T cells, leading to T-cell exhaustion and impaired antitumor immunity [39].

Collectively, these findings suggest the existence of an “immune suppressive 1q-signature” characterized by enhanced sSLAMF7 production, TAM expansion, exhausted T cells, and reduced NK-cell activity. This biological program may contribute to both treatment resistance and disease progression.

### 4.2. PBX1–FOXM1 Axis: A Central Driver of 1q+ MM

Recent multi-omics analyses have provided important mechanistic insights into why 1q gain confers aggressive clinical behavior and treatment resistance in MM [40]. Among the molecular pathways identified, the PBX1–FOXM1 signaling axis has emerged as a critical regulator of disease progression in 1q-positive MM. PBX1, a homeobox transcription factor encoded within the 1q region, is frequently overexpressed in MM cells harboring 1q gain. Integrated transcriptomic, epigenomic, and chromatin accessibility analyses have demonstrated that PBX1 induces the expression of FOXM1 and E2F family transcription factors, both recognized drivers of cellular proliferation and genomic instability.

Notably, while both FOXM1 and E2F2 expression correlate with adverse clinical outcomes, FOXM1 appears to play a unique role in 1q-positive MM. Comparative analyses have demonstrated preferential FOXM1 overexpression in MM cell lines harboring 1q gain. Functional studies further revealed that pharmacologic inhibition or genetic knockdown of FOXM1 selectively suppresses the growth of 1q-positive MM cells, indicating these cells develop a marked dependency on FOXM1 signaling for growth and survival [19,41].

**Figure 2:**
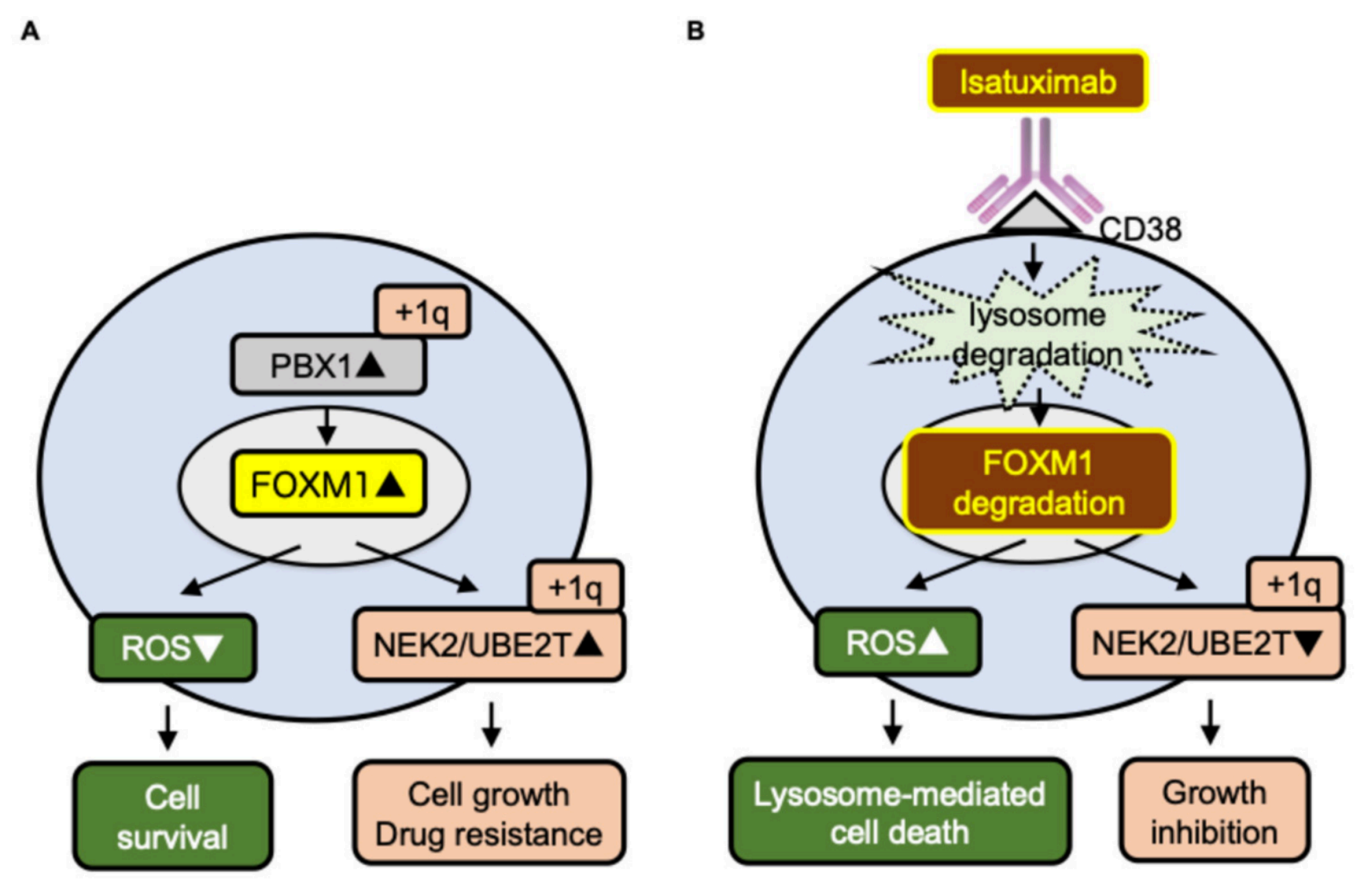
Biological consequences of 1q gain and the proposed mechanism of action of isatuximab in 1q-positive MM. (**A**) Proposed mechanisms by which 1q gain promotes MM cell proliferation and the acquisition of drug resistance. (**B**) Proposed mechanism of action of isatuximab against 1q gain-positive MM cells.

Mechanistically, FOXM1 regulates multiple downstream pathways associated with disease aggressiveness and therapeutic resistance. Chromatin immunoprecipitation sequencing (ChIP-seq) analyses have identified several FOXM1 target genes located within chromosome 1q, including NEK2 and UBE2T, both of which have been implicated in genomic instability, cell cycle progression, and resistance to anti-myeloma therapies [42,43]. These findings suggest the existence of a self-reinforcing oncogenic circuit in which 1q gain promotes PBX1 overexpression, leading to FOXM1 activation and subsequent induction of multiple resistance-associated genes (Figure 2A).

Importantly, the identification of FOXM1 dependency in 1q-positive MM provides a potential biological explanation for the differential activity of anti-CD38 antibodies observed in recent clinical studies. As discussed in the following section, emerging evidence suggests that isatuximab can suppress FOXM1 expression. This occurs through a unique mechanism involving antibody internalization and lysosomal disruption, thereby selectively targeting a critical vulnerability of 1q-positive MM cells.

## 5. A Potential Therapeutic Vulnerability in 1q+ MM

### 5.1. Isatuximab-Mediated FOXM1 Suppression

Among the mechanistic differences between daratumumab and isatuximab, the ability of isatuximab to directly induce MM cell death has attracted considerable interest. A recent study suggests this direct cytotoxic activity may be particularly relevant in MM cells harboring 1q gain. Although the molecular basis for this preferential activity remained unclear for many years, emerging evidence indicates that FOXM1 suppression may represent a key mechanistic event [18].

Isatuximab binding induces internalization of the CD38-antibody complex into MM cells. This process is substantially more pronounced than that observed with daratumumab and results in intracellular accumulation of the antibody within lysosomal compartments. In experimental models of 1q-positive MM characterized by high CD38 expression, isatuximab internalization was associated with significant cytotoxicity accompanied by marked downregulation of FOXM1 protein expression [18].

Importantly, FOXM1 suppression occurred at the protein level and was followed by loss of viability preferentially in 1q-positive MM cells. Given the established dependence of these cells on the FOXM1 signaling axis, these findings suggest that isatuximab directly targets a critical survival pathway of 1q-positive MM cells. Mechanistic investigations further suggest that lysosomal disruption may contribute to FOXM1 degradation. Following extensive antibody internalization, lysosomal membrane destabilization appears to promote the release of proteolytic enzymes into the cytoplasm, leading to degradation of FOXM1 protein [18] (Figure 2B).

FOXM1 suppression may also contribute to cell death by modulating oxidative stress. FOXM1 is known to regulate antioxidant defense pathways and limit intracellular reactive oxygen species (ROS) accumulation [44]. Consequently, loss of FOXM1 activity results in increased oxidative stress, mitochondrial dysfunction, and cell death. This mechanism may be particularly relevant in treatment-resistant MM cells, which rely heavily on FOXM1-mediated stress adaptation to survive therapeutic pressure (Figure 2A). Recent single-cell transcriptomic analyses have provided additional insights into the biology of MRD [45]. Residual myeloma cells harboring high-risk cytogenetic abnormalities exhibit significant enrichment of ROS-related signaling pathways, suggesting that enhanced antioxidant capacity and efficient ROS scavenging contribute to MRD persistence and therapeutic resistance. In this context, the ability of isatuximab to induce intracellular ROS accumulation may have particular therapeutic relevance. By disrupting ROS homeostasis through FOXM1 suppression and lysosome-associated cell death, isatuximab may preferentially eliminate MRD cells carrying double-hit high-risk cytogenetic abnormalities (Table 2). Although direct clinical evidence is currently lacking, this mechanism provides a biologically plausible explanation for the favorable outcomes observed in patients with double-hit disease treated with isatuximab-containing regimens in the IMROZ trial (isatuximab plus bortezomib, lenalidomide, and dexamethasone) [4]. Further mechanistic and translational studies are warranted to determine whether ROS modulation contributes to the enhanced efficacy of isatuximab in biologically high-risk MM.

The biological implications of FOXM1 suppression likely extend beyond direct cell killing. FOXM1-driven 1q-positive clones contribute to an immunosuppressive microenvironment characterized by elevated SLAMF7 expression, TAM expansion, T-cell exhaustion, and NK-cell dysfunction. Preferential elimination of these clones may therefore attenuate the broader “immune suppressive 1q-signature.” Thus, isatuximab may exert both tumor-intrinsic and microenvironmental effects through selective targeting of FOXM1-dependent disease biology.

This hypothesis may provide a biological explanation for several observations from recent clinical studies. In the ICARIA-MM trial (isatuximab plus pomalidomide [Poma] and dexamethasone) and IKEMA trials (isatuximab plus carfilzomib [CFZ] and dexamethasone), patients harboring 1q abnormalities derived substantial benefit from isatuximab-containing regimens [46]. Similarly, subgroup analyses from the IMROZ study suggested a clinical benefit in patients with particularly high-risk disease characterized by the coexistence of conventional high-risk cytogenetic abnormalities and 1q gain [4]. In addition, this mechanism may also contribute to prolonged second progression-free survival (PFS2) by facilitating durable eradication of biologically aggressive residual clones. While these findings should be interpreted cautiously due to the absence of direct comparative trials, they are consistent with the concept that isatuximab may preferentially target biologically aggressive 1q-positive MM.

### 5.2. CD56 as a Potential Determinant of Daratumumab Sensitivity

CD56 (neural cell adhesion molecule; NCAM) is expressed in most MM cells and has long served as a diagnostic marker in MM. Beyond its role as a phenotypic marker, emerging evidence suggests that CD56 may influence the biological response to anti-CD38 therapy [47]. Clinical analyses of RRMM patients receiving daratumumab-containing regimens have demonstrated superior PFS among patients with high CD56 expression. Notably, this association appears to persist even in subsets harboring 1q gain, suggesting that CD56 expression may partially overcome the adverse prognostic impact of 1q gain.

Mechanistic studies have provided a potential explanation for these observations. Following daratumumab binding, CD56-containing membrane complexes undergo internalization, resulting in downstream suppression of anti-apoptotic proteins, including MCL1 and BCL2. Because MCL1 is located within the amplified 1q region and represents a major survival dependency in 1q-positive MM [48], daratumumab-mediated downregulation of MCL1 may constitute an important mechanism of antitumor activity in this molecular subgroup (Table 2).

Unlike the FOXM1-centered mechanism proposed for isatuximab, the biological effects of daratumumab may therefore be mediated, at least in part, through disruption of MCL1-dependent survival pathways. This distinction highlights the possibility that the two anti-CD38 antibodies target different vulnerabilities.

## 6. Implications for B-Cell Maturation Antigen (BCMA)- and G Protein-Coupled Receptor Family C Group 5 Member D (GPRC5D)-Directed Immunotherapies

The therapeutic landscape of RRMM has been transformed by the introduction of T cell-redirecting therapies, including bispecific antibodies (BsAbs) targeting B-cell maturation antigen (BCMA) and G protein-coupled receptor family C group 5 member D (GPRC5D), as well as BCMA-directed chimeric antigen receptor (CAR) T-cell therapies [49]. Both teclistamab and talquetamab have demonstrated significantly prolonged PFS compared with conventional therapies in heavily pretreated patients with RRMM [50,51]. Nevertheless, a substantial proportion of patients eventually experience disease progression, highlighting the importance of understanding factors that influence the efficacy of these immune-based therapies.

Recent evidence suggests that both tumor burden and immune fitness play critical roles in determining response to T cell-redirecting approaches [52,53]. These observations raise an important question: can the biological differences between daratumumab and isatuximab influence the effectiveness of subsequent BCMA- or GPRC5D-directed therapies?

### 6.1. Soluble BCMA as a Biomarker of Tumor Burden and Therapeutic Resistance

One emerging mechanism of resistance to BCMA-targeted therapies involves soluble BCMA (sBCMA), which is generated through proteolytic cleavage of membrane-bound BCMA by γ-secretase [49]. Circulating sBCMA can bind BCMA-targeting antibodies, thereby reducing effective target engagement and creating a phenomenon often referred to as the “soluble BCMA sink.”

Clinical studies have demonstrated that lower baseline sBCMA concentrations are associated with superior responses to BCMA-directed therapies. Conversely, elevated sBCMA levels correlate with increased tumor burden and inferior treatment outcomes [54]. Because sBCMA is produced directly by MM cells, its concentration serves as a dynamic biomarker reflecting both disease burden and BCMA-related therapeutic accessibility [53,54,55].

Interestingly, genomic analyses have revealed an association between chromosome 1q abnormalities and elevated sBCMA levels. Patients harboring 1q gain frequently exhibit substantially higher circulating sBCMA concentrations than those without 1q abnormalities [54]. This observation suggests that 1q-driven disease may be intrinsically less favorable for BCMA-directed therapies because of both increased tumor burden and enhanced antigen sink effects.

### 6.2. Potential Advantages of Isatuximab Before BCMA-Directed Therapy

Within this framework, the ability of isatuximab to preferentially eliminate FOXM1-dependent 1q-positive clones may have implications beyond immediate disease control. Reduction of the 1q-positive tumor compartment could theoretically decrease total tumor burden, attenuate sBCMA production, and limit the development of the immune-suppressive microenvironment associated with the 1q signature.

Moreover, depletion of 1q-positive clones may reduce the prevalence of disease features associated with poor responses to T-cell–redirecting therapies, including T-cell exhaustion, TAM accumulation, and aggressive extramedullary progression. By targeting a molecularly defined high-risk population of MM cells, isatuximab-based therapy may therefore create a more favorable biological landscape for subsequent BCMA-directed treatment (Table 2).

MM cells express substantially higher levels of CD38 than normal immune cells [56]. Beyond its role as a surface antigen, CD38 functions as an ectoenzyme involved in NAD^+^ metabolism and extracellular adenosine generation. CD38 contributes to the production of immunosuppressive adenosine, which promotes signaling through the adenosine A2A receptor (A2AR). Activation of the adenosine–A2AR pathway has been implicated in the expansion and functional activation of Tregs, leading to the suppression of CTL activity and the establishment of an immunosuppressive tumor microenvironment [57]. Furthermore, adenosine signaling may contribute to the accumulation and functional polarization of TAMs. Clinically, an increasing disease burden has been associated with elevated circulating adenosine levels and the expansion of Treg populations in patients with MM [58,59].

Unlike daratumumab, isatuximab directly inhibits the enzymatic activity of CD38 [12]. Therefore, in addition to its direct cytotoxic effects and FOXM1 suppression, isatuximab may reduce extracellular adenosine production and attenuate adenosine-dependent immunosuppressive signaling. Theoretically, such effects could limit Treg activation, decrease TAM-promoting signals, and enhance CTL-mediated antitumor immunity. Consistent with this hypothesis, decreases in circulating Tregs have been reported in patients receiving isatuximab as monotherapy or in combination with dexamethasone. This suggests that inhibition of CD38 enzymatic activity may contribute to immune remodeling, in addition to direct antimyeloma effects [60]. Inhibition of CD38 enzymatic activity represents a unique biological property of isatuximab that may contribute to its immunomodulatory activity (Table 2).

We hypothesize that these combined effects—FOXM1 suppression, ROS-mediated cytotoxicity, and attenuation of immunosuppressive 1q-driven biology—may contribute to the durable disease control observed in some patients receiving isatuximab-containing regimens. This concept is supported by indirect evidence from clinical subgroup analyses; however, direct prospective evidence is currently lacking and remains an important area for future investigation. Future studies incorporating serial measurements of sBCMA, 1q clonal burden, and immune profiling will be required to determine whether early elimination of FOXM1-dependent clones improves responsiveness to BCMA-targeted therapies.

Recent clinical evidence further supports the importance of CD38-mediated purinergic metabolism in MM [61]. Analysis of paired bone marrow and peripheral blood samples from patients receiving daratumumab demonstrated a significant reduction in bone marrow adenosine concentrations following anti-CD38 therapy, confirming that CD38 blockade modulates purinergic metabolism in vivo. Nevertheless, adenosine concentrations remained above immunoregulatory thresholds at disease progression, suggesting persistent immune suppression despite sustained CD38 downregulation. Moreover, daratumumab-induced release of microvesicles carrying CD38 and other adenosinergic ectoenzymes (CD203a, CD73, CD26) may contribute to the maintenance of the immunosuppressive bone marrow niche during treatment resistance. These findings further emphasize the therapeutic importance of directly inhibiting CD38 enzymatic activity and targeting adenosine-mediated immune suppression.

These observations provide indirect support for the hypothesis that the stronger inhibition of CD38 enzymatic activity by isatuximab may confer additional immunological benefits beyond conventional Fc-mediated effector mechanisms. We emphasize, however, that direct clinical evidence comparing the immunomodulatory consequences of CD38 enzymatic inhibition between isatuximab and daratumumab is currently lacking. This hypothesis requires prospective investigation incorporating serial measurements of adenosine levels, Treg frequencies, and functional T-cell assays.

### 6.3. Daratumumab as an Immune-Priming Strategy for T-Cell Redirecting Therapies

While isatuximab may preferentially influence tumor burden and clonal composition, daratumumab may exert its greatest impact through immune remodeling.

Accumulating evidence indicates that successful responses to BsAbs are associated with specific immunologic characteristics, including increased numbers of CD3+ and CD8+ T cells, higher CD8/CD4 ratios, enrichment of effector-memory T cells, reduced frequencies of CD38-positive Tregs, and lower levels of T-cell exhaustion [53,62]. Notably, many of these features have also been observed following daratumumab treatment.

Multiple studies have demonstrated that daratumumab treatment is associated with depletion of CD38-positive Tregs, expansion of cytotoxic CD8+ T cells, increased T-cell clonality, and enrichment of effector-memory T-cell populations [63]. Importantly, these immunologic changes resemble several characteristics associated with favorable responses to BCMA- and GPRC5D-directed T cell-engaging therapies (Table 2).

Tunneling nanotubes (TNTs) are dynamic, actin-based membrane structures that enable direct communication between neighboring cells. These specialized conduits facilitate the transfer of mitochondria, proteins, nucleic acids, signaling molecules, and metabolic substrates across considerable distances within tissues [64]. In MM, stromal cells can donate functional mitochondria to MM cells through TNT, resulting in increased ATP production and induced resistance to therapeutic stress [65]. Recent studies in solid tumor models have demonstrated that mitochondrial transfer from tumor cells to T cells can promote T-cell dysfunction and exhaustion [66,67]. Although direct evidence in MM remains limited, similar mechanisms may contribute to immune suppression in advanced disease. The frequent enrichment of exhausted T cells in extramedullary lesions suggests that TNT-mediated metabolic reprogramming could play a role in establishing local immunosuppressive niches [68].

Importantly, CD38 appears to play a critical role in TNT formation and maintenance [58]. Among currently available anti-CD38 antibodies, daratumumab has been reported to inhibit TNT formation and mitochondrial transfer [69]. Although the precise molecular mechanisms remain incompletely defined, daratumumab-mediated disruption of CD38 signaling appears to interfere with the establishment of functional TNT networks. These findings suggest that daratumumab could reduce mechanisms of metabolic immune suppression and preserve T-cell functionality. Collectively, these effects suggest that daratumumab may act as an immune-conditioning agent capable of enhancing the efficacy of subsequent T cell-redirecting therapies (Table 2).

This concept is supported by emerging clinical observations demonstrating robust activity of teclistamab and talquetamab in patients previously exposed to daratumumab [52,70]. Although these findings should be interpreted cautiously because of patient selection and treatment heterogeneity, and the absence of randomized comparative data, they are consistent with the hypothesis that daratumumab-induced immune remodeling may improve the functional quality of residual T cells available for therapeutic redirection. We emphasize that this concept remains hypothesis-generating and requires validation in prospective biomarker-driven studies before informing clinical practice.

## 7. Efficacy in Extramedullary Disease (EMD)

EMD represents one of the most aggressive manifestations of MM and remains a major unmet need despite substantial therapeutic advances. EMD is characterized by the growth of MM cells outside the bone marrow and is associated with treatment resistance, rapid disease progression, and poor survival outcomes. Recent studies have provided important insights into the molecular mechanisms underlying the development of EMD, as well as the accompanying changes in tumor-intrinsic genetic alterations and the immune microenvironment [68,71,72]. However, the biological mechanisms underlying EMD are incompletely understood.

Several genomic studies have reported a high prevalence of 1q abnormalities in extramedullary lesions [73], supporting the concept that 1q-associated disease biology may contribute to extramedullary dissemination and progression. Recent immune profiling studies have further demonstrated that EMD is characterized by a profoundly immunosuppressive microenvironment [68]. Compared with intramedullary disease, extramedullary lesions contain increased numbers of exhausted T cells expressing inhibitory receptors and reduced frequencies of functional NK cells [72]. This immune landscape is consistent with the broader immune suppressive 1q-signature described above. Several features of EMD may compromise the efficacy of immune-mediated anti-CD38 mechanisms. Myeloma cells within extramedullary lesions frequently exhibit reduced CD38 expression, potentially limiting antibody binding and downstream effector functions [74]. Unlike daratumumab, isatuximab can induce direct cell death independently of immune effector cells. Consequently, its activity may be less dependent on the presence of functional NK cells or an intact immune microenvironment. Furthermore, preferential targeting of FOXM1-dependent 1q-positive clones may allow isatuximab to eliminate cellular populations that contribute disproportionately to EMD progression.

Conversely, several mechanisms may contribute to the reduced activity of daratumumab in EMD. The efficacy of daratumumab relies heavily on immune-mediated killing through ADCC and CDC, both of which may be compromised in extramedullary lesions. NK-cell depletion resulting from CD38-mediated fratricide may further reduce ADCC activity. Moreover, enrichment of exhausted T cells and immunosuppressive macrophages may limit the ability of the host immune system to cooperate with antibody-mediated tumor elimination. These biological factors may collectively diminish its effectiveness in advanced EMD.

Taken together, these findings suggest that EMD may represent a clinical setting in which the mechanistic differences between daratumumab and isatuximab become particularly important. The direct FOXM1-targeting activity of isatuximab may provide advantages in tumors driven by 1q-associated biology, whereas the immunomodulatory properties of daratumumab may be more relevant in disease settings where immune effector mechanisms remain intact (Table 3). Future studies specifically designed to evaluate anti-CD38 antibody activity in molecularly characterized EMD cohorts will be essential to determine whether these biological distinctions translate into clinically meaningful differences in patient outcomes.

## 8. Implications for Treatment Selection

The rapid expansion of therapeutic options in MM has transformed treatment decision-making from a simple choice between active agents to a complex process. This now requires consideration of disease biology, immune status, and future treatment opportunities. Although daratumumab and isatuximab are generally regarded as interchangeable anti-CD38 antibodies in current clinical practice, accumulating mechanistic evidence suggests they may exert distinct biological effects. These differences could influence optimal treatment sequencing.

Based on the available mechanistic and indirect clinical evidence, we propose a conceptual treatment-selection model integrating cytogenetic abnormalities, tumor-intrinsic vulnerabilities, and immune microenvironment characteristics (Table 3). This model is explicitly intended as a hypothesis-generating framework to stimulate future biomarker-driven prospective research and should not be interpreted as a clinical recommendation or validated treatment algorithm. Clinical decision-making regarding the choice between daratumumab and isatuximab should currently be based on approved indications, available randomized trial evidence, and individual patient characteristics, pending prospective validation of the biology-guided approach described herein. In particular, while approximately 35–50% of MM patients harbor 1q gain, we do not recommend isatuximab for all such patients; rather, we propose that 1q gain status may represent one of several biological factors to be considered in future prospective biomarker-driven treatment-selection studies.

### 8.1. Patient and Disease Characteristics

One major implication of recent biological studies is the recognition that 1q gain represents more than a prognostic marker. Instead, 1q gain appears to define a biologically distinct disease state characterized by activation of the PBX1–FOXM1 transcriptional program, enhanced expression of resistance-associated genes, increased SLAMF7 signaling, and establishment of an immunosuppressive microenvironment.

Within this context, isatuximab may offer unique advantages (Table 3). Through efficient antibody internalization and subsequent FOXM1 suppression, isatuximab appears capable of targeting a critical survival dependency of 1q-positive MM cells. Elimination of 1q-positive clones could theoretically attenuate the downstream consequences of 1q-driven disease biology, including tumor-associated macrophage expansion, T-cell exhaustion, NK-cell dysfunction, and extramedullary progression.

Accordingly, patients with 1q gain, particularly those with coexisting high-risk cytogenetic abnormalities, may represent a population in which isatuximab-based therapy warrants further investigation. Prospective validation will be necessary to determine whether biomarkers such as 1q gain, soluble SLAMF7, or soluble BCMA levels can identify patients most likely to benefit from this approach.

### 8.2. Optimizing the Tumor Microenvironment

In contrast, daratumumab may exert its greatest clinical impact through modulation of the immune microenvironment (Table 3). Beyond its direct antitumor activity, daratumumab promotes the depletion of regulatory immune populations, expansion of cytotoxic T cells, enhancement of T-cell clonality, and enrichment of effector-memory T-cell subsets. Emerging evidence also suggests a role in disrupting CD38-dependent tunneling nanotube networks, potentially limiting both metabolic support of tumor cells and immune exhaustion.

These effects may become increasingly important as T-cell–redirecting therapies move earlier in the treatment paradigm. The efficacy of BCMA- and GPRC5D-directed BsAbs and CAR-T therapies depends heavily on the presence of functional, non-exhausted T cells capable of sustained activation and proliferation. Consequently, daratumumab-mediated immune remodeling may provide benefits that extend beyond the period of active treatment.

This raises the possibility that daratumumab could function not only as an anti-myeloma therapy but also as an immune-conditioning strategy designed to enhance the effectiveness of future immunotherapies.

Regarding the optimal sequencing of daratumumab and isatuximab, prospective clinical evidence is currently limited. Based on the mechanistic framework described herein, a biology-guided approach may be considered: in patients with 1q gain or double-hit disease, isatuximab-based therapy administered earlier in the treatment course may preferentially eliminate FOXM1-dependent high-risk clones, potentially reducing tumor burden, attenuating soluble BCMA production, and limiting the immunosuppressive 1q signature, thereby creating a more favorable biological context for subsequent BCMA- or GPRC5D-directed therapies. Conversely, in patients in whom subsequent T cell-redirecting therapy is planned, daratumumab-based prior treatment may optimize the immune microenvironment through depletion of CD38-positive Tregs and disruption of tunneling nanotube networks. Published data suggest partial cross-resistance between daratumumab and isatuximab; patients progressing on one agent may still respond to the other, particularly when mechanisms of resistance differ. These considerations are incorporated into the proposed conceptual framework (Table 3) and await prospective clinical validation.

## 9. Conclusions

Daratumumab and isatuximab, while both targeting CD38, differ substantially in their mechanisms of action, particularly with respect to CDC activity, direct apoptosis induction, and modulation of the BMME. These mechanistic differences translate into distinct clinical profiles and opportunities for rational treatment selection. Further research into the cellular and molecular biology of CD38-mediated signaling and resistance mechanisms will be essential to optimize anti-CD38 therapy in MM.

The emergence of 1q gain, the PBX1–FOXM1 transcriptional axis, soluble SLAMF7-mediated immune suppression, and CD38-dependent immune remodeling has revealed previously unrecognized biological differences between daratumumab and isatuximab. These observations challenge the traditional view that anti-CD38 antibodies are largely interchangeable, suggesting that their optimal use may ultimately depend on specific molecular and immunologic disease contexts (Table 3).

The mechanistic framework proposed herein is explicitly hypothesis-generating and does not constitute a clinical recommendation for anti-CD38 antibody selection. Future prospective biomarker-driven studies are warranted to determine whether genomic alterations, such as 1q gain, and immune microenvironmental features can be integrated into the precise selection of anti-CD38 antibodies. Such biology-guided therapeutic strategies may represent an important step toward individualized treatment for MM.

## Figures and Tables

**Table 1:** Comparison of Key Features of Daratumumab and Isatuximab.

Feature	Daratumumab	Isatuximab
Target epitope on CD38	Unique epitope (C-loop region)	Distinct epitope (overlapping but different)
IgG subclass	IgG1κ	IgG1κ
CDC induction	Strong	Weak or absent
ADCC	Yes	Yes
ADCP	Yes	Yes
Direct cytotoxicity	Weak	Strong (via FOXM1 downregulation)
CD38 enzymatic inhibition	Weak	Yes
Administration	IV (weekly → biweekly → monthly)	IV (weekly → biweekly) or (weekly → biweekly → monthly)
Key approvals	NDMM + RRMM (multiple combos)	NDMM + RRMM (multiple combos)
Subcutaneous formulation	Yes (daratumumab SC)	Yes (isatuximab SC)

**Table 2:** Comparison of the Mechanistic and Therapeutic Differences Between Daratumumab and Isatuximab.

Feature	Daratumumab	Isatuximab
MRD depletion	VLA-4 down-regulation	ROS production
1q gain depletion	MCL1 down-regulation	FOXM1 down-regulation
Treg	Eradication	Inhibition
T-cell exhaustion	Inhibition	Inhibition
TAMs	(no data)	Inhibition

**Table 3:** Clinical Setting and Potentially Preferred Anti-CD38 Antibody (Biological Rationale).

Clinical Setting	Potentially Preferred Anti-CD38 Antibody	Biological Rationale
1q gain	Isatuximab	FOXM1 suppression
Double-hit	Isatuximab	ROS production
Persistent MRD	Daratumumab	VLA-4 down-regulation CAM-DR disruption
EMD	Isatuximab	Direct cytotoxicity
Before BsAbs	Daratumumab Isatuximab	Immune priming Tumor debulking

The treatment preferences listed in this table represent a hypothesis-generating mechanistic framework based on indirect biological and clinical evidence. They have not been validated in prospective randomized clinical trials and are not intended to guide clinical decision-making in the absence of prospective validation.

## Data Availability

No new data were created or analyzed in this study. Data sharing is not applicable to this article.
